# Raman Spectra of High-****κ**** Dielectric Layers Investigated with Micro-Raman Spectroscopy Comparison with Silicon Dioxide

**DOI:** 10.1155/2013/208081

**Published:** 2013-08-29

**Authors:** P. Borowicz, A. Taube, W. Rzodkiewicz, M. Latek, S. Gierałtowska

**Affiliations:** ^1^Institute of Electron Technology, Al. Lotników 32/46, 02-668 Warsaw, Poland; ^2^Institute of Physical Chemistry, Polish Academy of Sciences, Kasprzaka 44/52, 01-224 Warsaw, Poland; ^3^Institute of Microelectronics and Optoelectronics, Warsaw University of Technology, Koszykowa 75, 00-662 Warsaw, Poland; ^4^Institute of Physics, Polish Academy of Sciences, Al. Lotników 32/46, 02-668 Warsaw, Poland

## Abstract

Three samples with dielectric layers from high-**κ** dielectrics, hafnium oxide, gadolinium-silicon oxide, and lanthanum-lutetium oxide on silicon substrate were studied by Raman spectroscopy. The results obtained for high-**κ** dielectrics were compared with spectra recorded for silicon dioxide. Raman spectra suggest the similarity of gadolinium-silicon oxide and lanthanum-lutetium oxide to the bulk nondensified silicon dioxide. The temperature treatment of hafnium oxide shows the evolution of the structure of this material. Raman spectra recorded for *as-deposited* hafnium oxide are similar to the results obtained for silicon dioxide layer. After thermal treatment especially at higher temperatures (600°C and above), the structure of hafnium oxide becomes similar to the bulk non-densified silicon dioxide.

## 1. Introduction

The term high-**κ** means the dielectric material which has dielectric constant higher than silicon dioxide (SiO_2_). SiO_2_ is the most popular dielectric material used in technology of electronic devices due to the simple method of its production: surface oxidation. This manufacturing technique can be used in the case of circuits based on silicon or silicon carbide substrate. However, the application of SiO_2_ has a limitation that cannot be ignored. This limitation is leakage conductance [1]. The progress in miniaturization of electronic devices like transistor in central processing units (CPUs) implies the reduction of the thickness of dielectric layer. In the case of about 2 nm thick SiO_2_ film, the significant increase of leakage current is observed due to tunneling effect [2]. The increase of leakage current has negative influence on the electronic devices. The most important effect is the large increase of the power necessary to supply the devices. A significant part of this power is dissipated due to thermal effect, heating the device. The devices have to be efficiently cooled in order not to overcome the temperature limit of the thermal destruction. To sum up: further miniaturization of electronic devices requires dielectric materials with a larger dielectric constant than that of SiO_2_ [3].

Electric properties of dielectric layers depend on their molecular structures. An example of such dependence is the distribution of electric properties in MOS structures caused by distribution of mechanical stress [4]. The structure is reflected in vibrational spectra. Due to high precision, Raman spectroscopy can detect small deviations of molecular structure. The other advantage of this experimental technique is its nondestructive character. Examples of application of Raman spectroscopy for investigation of structural deviations are: analysis of mechanical stress distribution [5] and monitoring structural changes like densification caused by technological processes [6].

This work compares Raman spectra measured for three made from high-**κ** materials thin films deposited on silicon substrate. The first one, hafnium oxide, was already used for fabrication of CPU devices [7]. Two others, lanthanum-lutetium oxide and gadolinium-silicon oxide are candidates for application in electronic devices. Raman spectra of high-**κ** films are compared with data obtained for silicon dioxide layer.

## 2. Experimental

*Samples*. As a reference sample, Si wafer covered with SiO_2_ layer was used. Its manufacturing was already presented in the literature [8]. Hafnium oxide (HfO_2_) films were prepared with atomic layer deposition (ALD) technique. As substrates, four silicon (Si) wafers were used. The orientation of crystallographic axes was <100>. The wafers were covered with 6 nm thick base silicon dioxide film prior to deposition of HfO_2_. Three samples were subject to rapid temperature annealing (RTA) at 400°C, 600°C and 800°C. The fourth sample was used without thermal treatment (hereafter called *as-deposited*). Cooperation in respect to manufacturing of HfO_2_ samples was covered by the Institute of Electron Technology and Institute of Physics of Polish Academy of Sciences. Samples with gadolinium-silicon oxide (GdSiO) and lanthanum-lutetium oxide (LaLuO_3_) were manufactured at Gesellschaft für Angewandte Mikro- und Optoelektronik (AMO GmbH, Niemcy). In the case of GdSiO a two-step procedure was used. In the first step, Gd_2_O_3_ layer was deposited on Si substrate. In the second step, RTA was used to achieve GdSiO structure. RTA process was performed at 900°C during 60 s.

### 2.1. Apparatus

 The selection of excitation wavelength is of key importance for the Raman study of dielectric layers. In the case of visible excitation, the signal generated in thin dielectric layer can be even masked by multiphonon Raman scattering generated in Si substrate [9]. Due to this large background, Raman scattering is often treated as useless in the study of thin dielectric layers [10]. Deep-ultraviolet excitation significantly reduces the penetration depth of excitation light into the silicon substrate in comparison with standard visible excitation. The reduction of this penetration depth decreases the background intensity to negligible values. As a result, the Raman scattering from dielectric layer appears in the spectrum. Raman spectrometer used in this work had the same configuration as described in the literature [8]. For ellipsometry characterization, spectroscopic ellipsometer VASE (J. A. Woollam, USA) was used.

### 2.2. Data Analysis

 Mathematical pretreatment of the data included off-set and cosmic ray removal, baseline correction and intensity normalization. As a normalization condition, the intensity of one-phonon silicon line “520 cm^−1^” which is equal to 1 was chosen. The pretreatment was done with Grams 8 (Thermo Scientific, USA) program. The spectra measured for high-**κ** materials were compared with data obtained for SiO_2_ layer.

## 3. Results

### 3.1. Spectroscopic Ellipsometry

The following samples: Si/SiO_2_, Si/SiO_2_/HfO_2_ (*as-deposited*), Si/GdSiO and Si/LaLuO_3_ were characterized by means of spectroscopic ellipsometry prior to Raman investigation. Other samples containing HfO_2_ layer were too small for these measurements. The main features: refractive index for 300 nm and thicknesses of the samples, are collected in Table 1. The values of refractive index measured for thin silicon dioxide film are similar to the data of bulk material reported in the literature [11]. All high-**κ** materials have significantly larger optical density. The last column of Table 1 presents the thicknesses of the samples.

### 3.2. Raman Study

**Figure 1:**
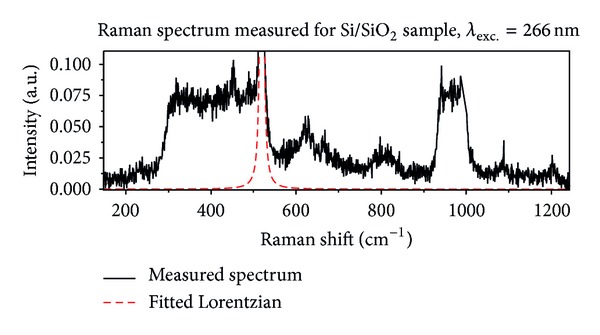
Raman spectrum measured for Si/SiO_2_ sample, excitation wavelength 266 nm. Black solid line represents measured data; dashed red line represents fitted Lorentzian profile modeling one-phonon Si line.

Figure 1 presents the data collected for Si/SiO_2_ sample. Black solid line marks measured spectrum and red dashed line designates fitted Lorentzian profile modeling one-phonon silicon line. The following bands can be recognized in the spectrum (except Si line “520 cm^−1^”):

- weak band with maximum at about 230 cm^−1^;
- relatively strong band spread from 300 cm^−1^ to one-phonon Si line (~550 cm^−1^);
- band with two maxima at about 630 cm^−1^ and 670 cm^−1^;
- broad band with maximum at about 810 cm^−1^;
- strong band spread from 930 cm^−1^ to 1030 cm^−1^;
- two weak bands with maxima at about 1090 cm^−1^ and 1200 cm^−1^.

**Figure 2:**
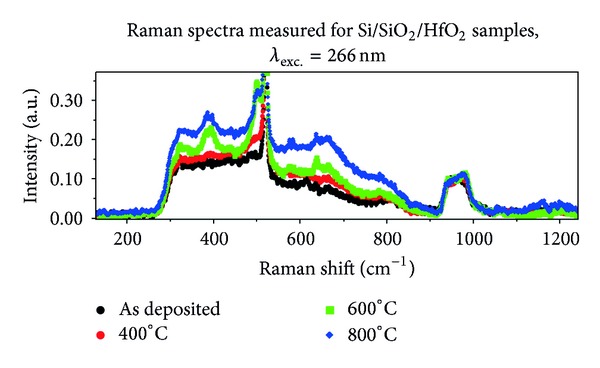
Raman spectra measured for Si/SiO_2_/HfO_2_ sample, excitation wavelength 266 nm. Black solid line represents *as-deposited* sample, red points represents sample annealed at 400°C, green points represents sample annealed at 600°C, blue points represents sample annealed at 800°C.

Raman spectra measured for silicon wafers covered with HfO_2_ are presented in Figure 2. Raman spectra recorded for HfO_2_ are similar to the data measured for SiO_2_ layer. The two most important common features observed for both dielectric materials can be recognized without detailed analysis:

- the absence of the so-called boson band;
- the presence of the band between 930 cm^−1^ and 1030 cm^−1^ assigned in the literature to multi-phonon scattering generated in Si substrate [9].

The intensity of the Raman scattering observed for HfO_2_ layer is 2 ÷ 3 times larger than the intensity recorded for SiO_2_ film. This comparison is done for normalized spectra. Both samples, Si/SiO_2_ and Si/SiO_2_/HfO_2_, have two internal standards: line “520 cm^−1^” and the multi-phonon band placed between 930 cm^−1^ and 1030 cm^−1^. Normalization of the spectra for both standards gives similar, relation between intensities of the bands assigned to vibrations in dielectric materials.

**Figure 3:**
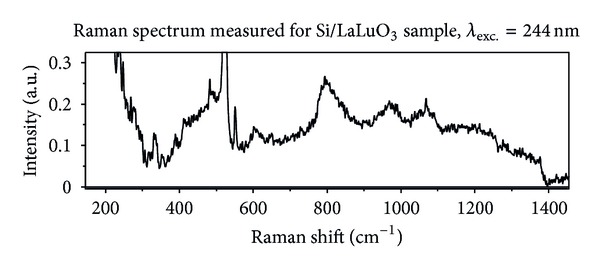
Raman spectrum measured for Si/LaLuO_3_ sample, excitation wavelength 244 nm.

**Figure 4:**
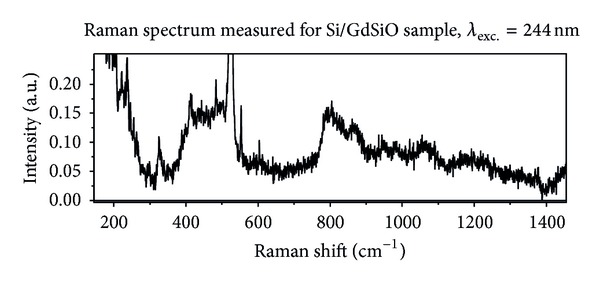
Raman spectrum measured for Si/GdSiO sample, excitation wavelength 244 nm.

Figures 3 and 4 present Raman spectra measured for LaLuO_3_ and GdSiO, respectively. In both cases excitation with second harmonic of Ar^+^ line 488 nm (*λ* = 244 nm) was used. Both spectra are similar. The intensities of the Raman scattering recorded for LaLuO_3_ and GdSiO are about twice larger in comparison with the signal coming from SiO_2_ film. Since the spectra observed for LaLuO_3_ and GdSiO are similar their common features will be discussed together (see Section 4).

## 4. Discussion

Let us start from short analysis of Raman spectrum recorded for reference sample—Si/SiO_2_ (Figure 1). The band placed between 930 cm^−1^ and 1030 cm^−1^ is assigned to multi-phonon scattering generated in Si substrate [9]. The other bands listed in Section 3 can be assigned to vibrations in SiO_2_. The SiO_2_ layer has a noncrytalline structure [12]. It can contain small area of quasi-crystalline form [12] like cristobalite, coesite, or crystalline quartz [13]. The area with amorphous densified structure [14, 15] can also appear in the SiO_2_ layer [12]. Taking into account data available in the literature the assignment of the observed bands to the oscillation in silicon dioxide can be done. However, one should take into account that the data reported in the literature was measured for bulk material and excitation in visible spectral range. The band with maximum at 230 cm^−1^ can be correlated with scissoring in [SiO_4/2_] tetrahedron [16] or with strong line of cristobalite which has the maximum for the same value of Raman shift [13]. The main band placed between 300 cm^−1^ and 550 cm^−1^ can be a combination of several bands. The following SiO_2_ vibration can contribute to this band:

- scissoring in extended tetrahedron [SiO_4/2_]-[Si_4/4_] labeled by D_3_ [16];
- bending in rings with a number of elements equal or larger than 5 (5+ rings) labeled by D_4_ [16];
- bending in Si-O-Si bridges labeled by R [16];
- vibration associated with four-member rings, so-called defect band, labeled by D_1_ [16].

Strong lines from crystalline forms of SiO_2_ are also placed in the range of Raman shift between 300 cm^−1^ and 550 cm^−1^ [13]. However, the contribution of the crystalline structures is so small that their intensities do not exceed the signal-to-noise ratio [12]. D_1_ band has the maximum at about 490 cm^−1^ in non-densified structure and at about 520 cm^−1^ in densified structure [6, 12, 14]. This band is placed so close to the one-phonon Si line that it is impossible to recognize it without mathematical analysis of the spectrum. The densification of SiO_2_ layer is reflected in the shape and position of the next two bands having maxima at about 620 cm^−1^ and 800 cm^−1^ [6, 14, 15]. The band with the maximum at about 670 cm^−1^ can be correlated with strong line reported for coesite and having the maximum at 661 cm^−1^ [13]. The bands placed around 1090 cm^−1^ and 1200 cm^−1^ are assigned to Si-O stretching vibration [14]. The last important feature of Raman spectrum recorded for SiO_2_ thin film is the absence of boson band observed for visible excitation in the case of bulk material [14]. It should be emphasized that boson band was not observed for bulk SiO_2_ in the case of deep ultraviolet excitation [8].

The bands observed for HfO_2_ and placed above 1000 cm^−1^ are much weaker than similar bands in SiO_2_ spectrum. The most important difference between Raman scattering observed for silicon dioxide and hafnium oxide is the shape of the spectrum in the range between 300 cm^−1^ and 930 cm^−1^. In the case of SiO_2_ three separate bands can be recognized in the spectrum: the main band placed between 300 cm^−1^ and 550 cm^−1^, D_2_ band placed around 620 cm^−1^ and a band “800 cm^−1^”. In the spectrum observed for HfO_2_ film the bands are merged in such a way that the main band placed between 300 cm^−1^ and 550 cm^−1^ has a tail extended to 930 cm^−1^. In the case of the spectrum observed for the sample, *as-deposited* weak band placed around 800 cm^−1^ can be recognized on this tail. The range 600 cm^−1^–700 cm^−1^ contains no unambiguous band. The main band placed between 300 cm^−1^ and 550 cm^−1^ is similar for spectra recorded for SiO_2_ and HfO_2_ *as-deposited* films.

Let us compare Raman spectra of HfO_2_ recorded film after thermal treatment. In the case of the sample annealed at 400°C, only a small increase of the main band intensity is observed. No changes in the shape of the spectrum appear. Annealing at higher temperature (600°C and 800°C) leads to a significant increase of the Raman scattering intensity combined with changes of the shape of the spectrum. The following bands (maxima) appear in the spectra of Si/SiO_2_/HfO_2_ samples annealed at 600°C and 800°C: 320 cm^−1^, 380 cm^−1^, 600 cm^−1^, 670 cm^−1^. An additional relatively narrow band appears near the Si one-phonon line. The band is merged with “520 cm^−1^” line, and its maximum is placed at about 490 cm^−1^. The weak band placed around 800 cm^−1^ becomes stronger, and its maximum shifts towards smaller values of Raman shift by about 10 cm^−1^. The following analogies can be drawn between bands observed for annealed HfO_2_ film and the data reported for bulk SiO_2_ in the literature:

- band with the maximum at 320 cm^−1^ corresponds to D_3_ band assigned to scissoring in extended tetrahedron [SiO_4/2_]-[Si_4/4_];
- band with the maximum at 380 cm^−1^ corresponds to D_4_ band assigned to bending in 5+ rings in SiO_2_;
- band with the maximum at 430 cm^−1^ corresponds to R band assigned to bending in Si-O-Si bridges;
- band with the maximum at 490 cm^−1^ corresponds to D_1_ defect band assigned to vibrations in 4-members rings in SiO_2_ structure;
- band with the maximum at 600 cm^−1^ corresponds to D_2_ defect band assigned to vibrations in 3-member rings in SiO_2_ structure;
- band with the maximum at 800 cm^−1^ corresponds to complex so-called “800 cm^−1^” band in SiO_2_ structure [14].

The positions and shapes of Raman band equivalents of D_1_, D_2_ and “800 cm^−1^” recorded for the samples Si/SiO_2_/HfO_2_ are similar to the data reported for non-densified bulk SiO_2_. In the case of silicon oxide layer, bands D_2_ and “800 cm^−1^” suggest densified character of the film.

The first feature that appears in spectra recorded for LaLuO_3_ and GdSiO is the significant signal below 300 cm^−1^. It corresponds to boson band reported for excited with visible light Raman scattering from bulk SiO_2_ [14]. The absence of the relatively strong band in the range of Raman shift between 930 cm^−1^ and 1030 cm^−1^ assigned to second order scattering in Si substrate is also observed for both dielectric layers (LaLuO_3_ and GdSiO). Small differences between Raman spectra of LaLuO_3_ and GdSiO in this range of Raman shift will be discussed later. Spectra recorded for LaLuO_3_ and GdSiO contain not only broad bands, but also narrow lines. full width at half maximum (FWHM) of these lines is the order of 1 cm^−1^. It suggests vibrations of crystalline structures as origin of these lines. Amorphous structures generate bands with FWHM at the order of 10 cm^−1^. The first line appearing in the scattering observed for both dielectrics has the maximum at about 330 cm^−1^. This line has the best correlation with two strong lines reported for tridymite. These lines are centered around 320 cm^−1^ and 355 cm^−1^ [13]. Broad band merged with one-phonon Si line starts between 380 cm^−1^ and 400 cm^−1^ and is significantly narrower in comparison with the main band observed for SiO_2_ layer. One narrow line appears in the range of Raman shift between 380 cm^−1^ and 540 cm^−1^ against the background of the main broad band. The maximum of this line equals 390 cm^−1^ for LaLuO_3_ and 420 cm^−1^ in the case of GdSiO. The line appearing within broad band has the best correlation with two strong lines of tridymite. The reported maxima positions of tridymite lines are equal to 403 cm^−1^ and 422 cm^−1^ [13]. The main broad band has different shapes for LaLuO_3_ and GdSiO. In the case of LaLuO_3_ (Figure 3) the fast increase of the band intensity is observed for Raman shift in the range 380 cm^−1^–420 cm^−1^. It is followed by lower increase in the range 420 cm^−1^–500 cm^−1^. In the range of Raman shift between 500 cm^−1^ and 540 cm^−1^ one-phonon Si line dominates in Raman spectrum. One narrow line with the maximum at 480 cm^−1^ and slightly broader band with the maximum at 490 cm^−1^ appear in LaLuO_3_ spectrum. Narrow line can be compared with following lines reported for crystalline structures of SiO_2_: tridymite (457 cm^−1^), crystalline quartz (465 cm^−1^), or moganite (501 cm^−1^) [13]. Broader band has the same position as D_1_ reported for non-densified form of bulk SiO_2_ [16]. In the case of GdSiO the fast increase of the main band intensity is observed in the range of Raman shift 380 cm^−1^–400 cm^−1^. Except previously discussed narrow line with the maximum at 420 cm^−1^ two band with maxima at 430 cm^−1^ and 490 cm^−1^ can be recognized in the spectrum. The band with the maximum at 430 cm^−1^ has the best correlation with band R reported for SiO_2_ spectrum. Band with the maximum at 490 cm^−1^ has the best correlation with D_1_ band reported for non-densified bulk silicon oxide. Appearing in LaLuO_3_ spectrum line with the maximum at 480 cm^−1^ has very small FWHM. Narrow line with the maximum at about 550 cm^−1^ appears in Raman spectra of both oxides: LaLuO_3_ and GdSiO. The FWHM of the line observed for LaLuO_3_ is significantly smaller than the FWHM of the same line in GdSiO spectrum. The line 550 cm^−1^ has the best correlation with coesite line centered around 522 cm^−1^ [13]. The next two bands appearing in Raman spectra of lanthanum-lutetium oxide and gadolinium-silicon oxide have the maxima at 600 cm^−1^ and 640 cm^−1^. The bands can be correlated with D_2_ band reported for silicon oxide. The maximum 600 cm^−1^ corresponds to non-densified structure, and 640 cm^−1^ corresponds to densified one. The next band present in Raman spectra recorded for lanthanum-lutetium and gadolinium-silicon oxides has the maximum at about 800 cm^−1^. The position of the maximum and asymmetric shape of the band with steep gradient on the blue side of maximum (for smaller values of Raman shift) look like the band “800 cm^−1^” observed for non-densified bulk SiO_2_. The intensity of the band refering to the intensity of one-phonon Si line in the case of LaLuO_3_ and GdSiO is about twice larger than for silicon dioxide layer. Also the intensity ratio of the following bands “800 cm^−1^” and main band (placed below Si line “520 cm^−1^”) is in the case of LaLuO_3_ and GdSiO few times larger than for SiO_2_. In the range of Raman shift between 930 cm^−1^ and 1030 cm^−1^ the following features can be recognized:

- single asymmetric band with the maximum at 970 cm^−1^ for LaLuO_3_;
- two band with maxima at 950 cm^−1^ and 980 cm^−1^ for GdSiO.

Raman spectra recorded for both oxide layers, LaLuO_3_ and GdSiO, show broad, oval background which ranges from 650 cm^−1^ to 1400 cm^−1^. Bands described previously appear on this background. The Raman signals observed for LaLuO_3_ and GdSiO do not have typical shape of the band observed for SiO_2_ layer and are assigned to multi-phonon scattering from Si substrate [9]. It is possible that the band assigned to multi-phonon scattering is modified and partially masked by the broad background. The last two bands which can be recognized in spectra measured for lanthanum-lutetium and gadolinium-silicon oxide films have the maxima at about 1070 cm^−1^ and 1200 cm^−1^. The band with the maximum at 1070 cm^−1^ has a symmetric shape. The band with the maximum at 1200 cm^−1^ seems to be asymmetric. The tail of this band ranges up to 1400 cm^−1^. The bands with maxima at 1070 cm^−1^ and 1200 cm^−1^ can be correlated with bands reported for silicon oxide and assigned to the longitudinal optical (LO) and transverse optical (TO) pair of Si-O stretching vibrations [14, 17, 18]. Maxima of these bands reported in the literature are equal to about 1075 cm^−1^ and 1200 cm^−1^, respectively.

## 5. Conclusions

In earlier work, the usefulness of deep-ultraviolet Raman spectroscopy in investigation of dielectric layers in silicon based circuits was presented [8]. In this work, application of this excitation to high-**κ** materials was discussed. Raman spectra of three high-**κ** materials, in particular, hafnium oxide, lanthanum-lutetium oxide and gadolinium-silicon oxide were compared with silicon dioxide. All investigated materials can be divided into two groups. The first one consists of silicon oxide and hafnium oxide, and the other one consists lanthanum-lutetium oxide and gadolinium-silicon oxide. Inside each group, observed Raman spectra are similar. In the case of first group, this similarity concerns spectra of SiO_2_ and HfO_2_ *as-deposited*. The bands observed for high-**κ** materials can be correlated with bands recorded for silicon oxide. Analysis of spectra obtained for HfO_2_ annealed at the temperature 600°C or higher as well as for LaLuO_3_ and GdSiO suggests similarity of these materials with non-densified structure bulk SiO_2_. The structure of following layers: SiO_2_, HfO_2_ *as-deposited* and HfO_2_ annealed at 400 °C seems to be similar to densified bulk silicon dioxide.

The intensity of Raman scattering generated by hafnium oxide, lanthanum-lutetium oxide, and gadolinium-silicon oxide layers is 2-3 times higher than scattering from silicon oxide layer. The comparison is done for intensities related to the intensity of one-phonon line from silicon substrate.

The last point to sum up is the behavior of Raman spectra in the range 930 cm^−1^–1030 cm^−1^. In the case of HfO_2_ and SiO_2_ layers, the band appearing in this range is similar to the data reported for multi-phonon scattering from silicon. For LaLuO_3_ and GdSiO, bands in this range of Raman shift are mixed with broad and oval background ranging from 650 cm^−1^ to 1400 cm^−1^. This background can modify the shape of observed bands.

## Figures and Tables

**Table 1:** Refractive indices obtained for excitation wavelength equal to 300 nm and film thicknesses measured by means of spectroscopic ellipsometry for silicon dioxide, hafnium oxide, gadolinium-silicon oxide, and lanthanum-lutetium oxide.

Dielectric layer	*n* (*λ* = 300 nm)	*d* [nm]
SiO_2_	1.495 ± 0.005	64 ± 2
HfO_2_ (“as-deposited”)	2.109 ± 0.001	46 ± 2
GdSiO	1.954 ± 0.004	47.5 ± 0.5
LaLuO_3_	2.077 ± 0.001	8.0 ± 0.3
